# Does Gender Matter? Female Representation on Corporate Boards and Firm Financial Performance - A Meta-Analysis

**DOI:** 10.1371/journal.pone.0130005

**Published:** 2015-06-18

**Authors:** Jan Luca Pletzer, Romina Nikolova, Karina Karolina Kedzior, Sven Constantin Voelpel

**Affiliations:** 1 Focus Area Diversity, Jacobs University, Bremen, Germany; 2 Social and Organizational Psychology, VU University Amsterdam, Amsterdam, The Netherlands; 3 KU Leuven, Leuven, Belgium; 4 Bremen International Graduate School of Social Sciences (BIGSSS), Jacobs University Bremen, Bremen, Germany; 5 Institute of Psychology and Transfer, University of Bremen, Bremen, Germany; Cinvestav-Merida, MEXICO

## Abstract

In recent years, there has been an ongoing, worldwide debate about the representation of females in companies. Our study aimed to meta-analytically investigate the controversial relationship between female representation on corporate boards and firm financial performance. Following a systematic literature search, data from 20 studies on 3097 companies published in peer-reviewed academic journals were included in the meta-analysis. On average, the boards consisted of eight members and female participation was low (mean 14%) in all studies. Half of the 20 studies were based on data from developing countries and 62% from higher income countries. According to the random-effects model, the overall mean weighted correlation between percentage of females on corporate boards and firm performance was small and non-significant (*r* = .01, 95% confidence interval: -.04, .07). Similar small effect sizes were observed when comparing studies based on developing vs. developed countries and higher vs. lower income countries. The mean board size was not related to the effect sizes in studies. These results indicate that the mere representation of females on corporate boards is not related to firm financial performance if other factors are not considered. We conclude our study with a discussion of its implications and limitations.

## Introduction

Advancing gender equality and female representation in corporate governance has increasingly become the focus of societal and political debates in various countries [1]. Despite extensive efforts to increase women’s presence on corporate boards, men still dominate the corporate world. The financial effects of increased female representation on corporate boards may crucially determine if, and how, regulations to promote females to higher positions are implemented, because pursuing financial success is an innate characteristic of every company. While a number of scientific studies have investigated the relationship between gender diversity and firm financial performance, their conclusions are equivocal [2], [3]. These empirical discrepancies have led to a lack of conclusive evidence about the relationship between increased female representation and firm performance, creating uncertainty for policy makers, CEOs, and investors around the world. Owing to the conflicting evidence from primary studies, systematically summarizing the existing data on the topic in a quantitative meta-analysis has merit. While our general research question is similar to that of Post and Byron [4], the methodological and analytical approach differs substantially between the two analyses. Our study aims to investigate the relationship of interest with a different, more rigorous and controlled methodological approach, and subsequently compares the results of the two meta-analyses. Investigating this relationship in a different sample and with different operationalizations of the variables (compared to Post and Byron [4]) is especially important, because, in their analysis, the overall mean weighted correlation between female participation on boards and firm performance was very small (only marginally different from zero). Thus, this paper investigates the general relationship between female representation and firm performance using a new and different methodological approach, highlights our additional contribution to the literature, and compares the similarities and differences between the two analyses.

### Literature Overview

A board of directors monitors the activities of an organization or company. It sets the corporate strategy, appoints and supervises senior management, and functions as the main corporate governance mechanism. The role of the board in determining the corporate strategy therefore influences firm performance. Since diversity is often considered a double-edged sword (e.g., [5]), meaning that increased diversity can result in advantages and disadvantages regarding desired outcomes, a board composed of diverse directors affects firm performance either positively or negatively. Diversity’s positive and negative effects could also neutralize each other, or could depend on how it is managed [6]. Along these lines, a meta-analysis by Webber and Donahue [3] examined the effects of diversity on work group performance in a sample of 45 effect sizes. Low job-related (e.g., age, gender) and highly job-related diversity (e.g., educational background) were measured, but both failed to show a significant relationship with work group performance.

Further, primary studies also do not show a clear consensus on whether gender diversity benefits or disadvantages firm performance [7], [8]. At first glance, the relationship between female representation on corporate boards and firm financial performance shows a similar pattern to that of the general diversity-performance relationship, being either positive [9], negative [10], or non-significant [11]. Thus, it remains unclear if increased female representation on corporate boards is associated with firm performance, and, if so, in which direction.

Advocates of greater female representation on corporate boards usually rely on two lines of arguments: the ethical or the business case for diversity [12]. The former argues that women should be considered for leadership positions for equality reasons. The aim is therefore not directly to increase performance, but rather that greater female representation is considered a positive and just result in itself [13]. Thus, a higher proportion of females on boards might not necessarily be related to better firm performance, but would reflect that boards with more females closely represent the ‘real world’, while other factors than gender alone contribute to better financial outcomes. The business case for diversity holds that if a board comprises heterogeneous directors, diversity leverages financial growth and success [12], indicating that a higher proportion of females could be related to better firm performance. A final outcome, not explained by either of the cases above, would be a negative relationship between a higher proportion of females on boards and lower firm performance. The aim of this article is to summarize the already existing quantitative evidence and attempt to explain the results in light of these cases.

### Positive Effects of Increased Female Representation on Firm Performance

The *business case* for diversity holds that diverse team members improve corporate governance by introducing broader knowledge bases and experiences [12], [14]. Accordingly, the *cognitive resource model* suggests that as (gender) diversity in groups increases, the available cognitive resources increase as well [15], [16]. If used effectively, these diverse perspectives can contribute to a more thorough search for alternative solutions to problems because they introduce new perspectives to the boardroom [17]. These diverse perspectives also foster a critical analysis of complex problems, prevent premature decision-making [5], [6], [18], [19], and develop creative and innovative solutions [20]. Hence, increased female representation on corporate boards should improve firm financial performance through the diverse perspectives introduced to the boardroom.

Another essential argument in support of the business case for diversity is that women introduce useful female leadership qualities and skills to the boardroom. These include, for example, risk averseness and less radical decision-making [21], [22], as well as more sustainable investment strategies [23]. In addition, female leaders fulfill their leadership roles in a more transformational way than their male counterparts, distinguishing themselves especially through their encouraging and supportive treatment of colleagues and subordinates (i.e., individualized consideration; [24]). Females are also said to value their responsibilities as directors higher, which is associated with more effective corporate governance [25]. Furthermore, diversity on corporate boards generally benefits organizations, by providing wider and better connections and ties to suppliers, organizations, and consumers, which decrease market uncertainties and dependencies [8]. In sum, an increased female presence on corporate boards is associated with the introduction of new desirable leadership skills and a variety of strategic advantages for companies. Following this reasoning, we expect a positive relationship between increased female representation and firm financial performance.

### Negative Effects of Increased Female Representation on Firm Performance

Individuals are likely to perceive others and themselves in terms of salient social categories, such as gender, thereby creating in- and out-groups [26]. These categorization tendencies, which might lead to heightened gender salience and a perceived lack of alignment with the group’s stereotypes [27], can compromise functional team processes when demographic subgroups emerge. If the emerging subgroups on corporate boards are based on gender, communication and cooperation might be impaired [6], leading to increased conflicts between board members. The probability of conflict might be further enhanced if the directors identify stronger with the opinions of fellow directors of the same gender [28], or if the introduction of new perspectives, previously mentioned as one of diverse groups’ advantages, backfires [29]. In turn, this potential for interpersonal conflicts might retard the decision-making process and lead to a lack of cohesion between board members and to decreased strategic consensus [30], [31], hindering corporate boards’ effectiveness. In fast-paced environments, such as on corporate boards where strategic decisions need to be taken quickly, conflict-free communication is crucial to maintain effective performance [32]. And even if these issues can be overcome, the additional time and resources spent on solving them might decrease group and organizational performance [33].

These far-reaching potentials for impaired team processes might especially challenge females, who struggle to participate and maintain their standing in the already male-dominated boardroom [34] and are at risk of experiencing role ambiguity and role conflict, because they do not conform to typical gender roles in leadership [35]. Such females might be perceived as “tokens” to meet society’s expectations or those of important stakeholders, and could therefore be marginalized and not be taken seriously on the board [36], which might subsequently hinder their and the entire board’s performance [37], [38].

All in all, increased female representation could potentially lead to decreased firm financial performance due to a number of strategic disadvantages, increased interpersonal conflicts, and their associated negative consequences. Following this reasoning, we expect a negative relationship between increased female representation and firm financial performance.

In conclusion, it is difficult to determine the relationship between female representation on boards and firm performance a priori. Summarizing all studies measuring the relationship between female representation on corporate boards and firm financial performance could provide substantive evidence to address the question whether increased female representation on corporate boards alone is positively or negatively related to firm financial performance. The meta-analysis by Post and Byron [4] provided the first systematic summary of this relationship in studies selected from a range of electronic databases and unpublished sources. Using 140 studies (92 published, 48 unpublished), they show that the relationship is very small (*r* = 0.047 between female representation and accounting returns, and *r* = 0.014 between female representation and market performance), and that it might depend on moderators, such as shareholder protection or gender parity in a given country. As opposed to Post and Byron [4], our study follows a more rigorous and controlled methodological approach by investigating the relationship between *percentage of females* on corporate boards and firm financial performance, operationalized as *return on assets* (*ROA*), *return on equity* (*ROE*), and *Tobin’s Q* (*Q*), by means of a meta-analysis of articles published in peer-reviewed academic journals. These narrow operationalizations of the variables could increase the certainty with which theoretical and practical implications can be deduced. In light of the study by Post and Byron [4], we also aim to compare the results of the two meta-analyses. A systematic investigation of the published literature could also reveal potential moderators of such a relationship based on systematic differences between various studies in terms of their source of data and company characteristics, which could only be determined post hoc once we had located and coded all the available data. Based on the studies reviewed above and the meta-analysis of Post and Byron [4], we hypothesized that female representation on corporate boards is either positively or negatively related to firm financial performance, but that the magnitude of such a relationship is likely to be small.

## Methods

### Systematic Search Strategy

A systematic literature search was conducted in EBSCO on March 7, 2014, using the search strategy described in Table 1. The databases and the search terms we utilized differ from those used by Post and Byron [4]. Therefore, the two meta-analyses are based on a different sample of articles. In addition, we conducted a hand-search of the reference sections of review articles in this field and of Google Scholar, using the terms listed in Table 1 between March 7 and May 12, 2014. Unlike Post and Byron [4], we only searched for (and included) studies published in peer-reviewed academic journals.

**Table 1:** Search terms and databases (all searches conducted in English).

Number of sources (*k*)	Search terms and limits	Databases (1986-March 2014)
325	[Subject OR Title (“gender diversity” OR gender OR female OR wom*n OR "board diversity" OR "board of director*" OR "board structure")] AND [Subject OR Title (“organi*ation* performance” OR “firm performance” OR "financial performance" OR “company performance”)]; limits: Academic Articles, English Language	PsycInfo; EconLit; Business Source Premier; Academic Search Premier

### Study Selection

**Fig 1:**
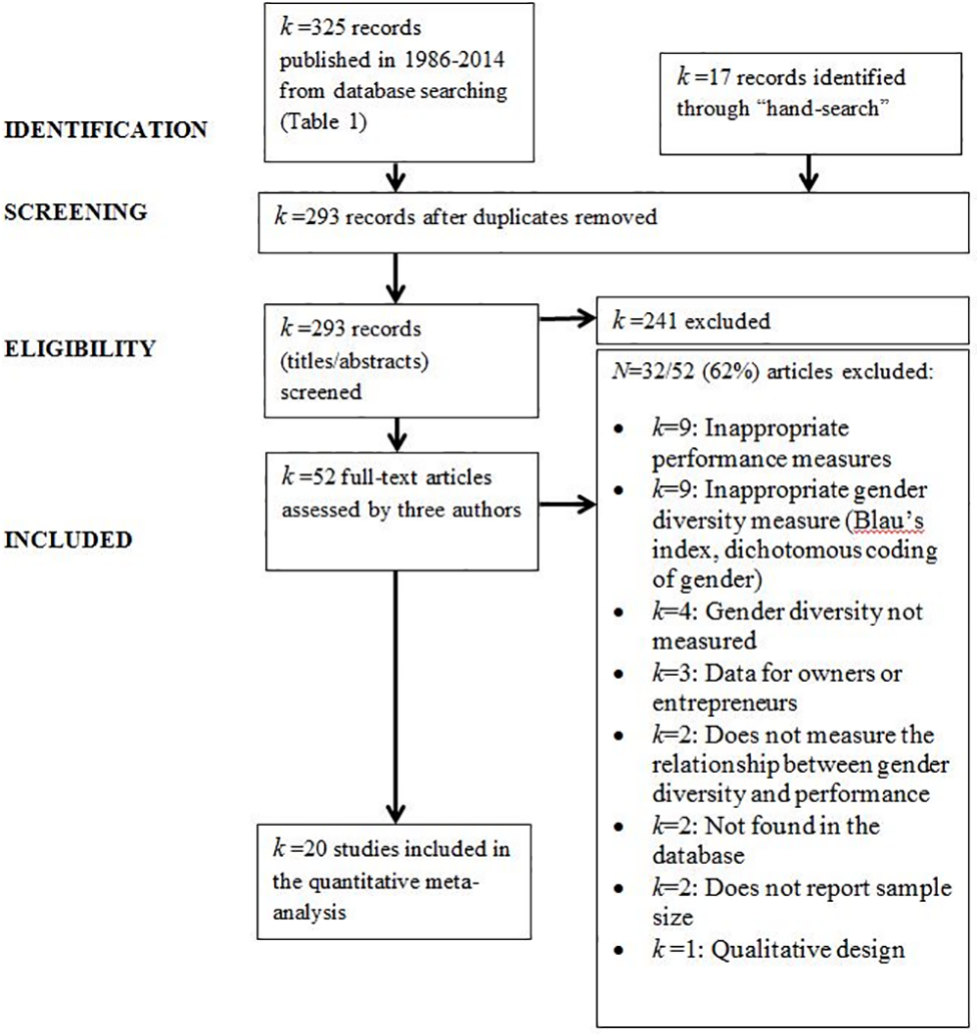
Study assessment and exclusion criteria. *k* = number of studies.

The study selection process is summarized on the PRISMA flowchart [39], Fig 1.

Of the 325 sources identified during the electronic search (Table 1) and the 17 sources identified through the hand search, 52 studies met the inclusion criteria and were fully examined (Fig 1). The studies selected for the final meta-analysis had to have a quantitative design and report the Pearson product moment correlation coefficient, *r*, between the percentage female representation on boards of directors and firm performance (measured as *ROA*, *ROE*, or *Q*), with the number of observations (number of firms × total length of data collection in years) used as the sample size. Studies not reporting the correlation coefficient, but including the variables required in our analysis, were also included in our sample if the authors (contacted via email) provided these correlations. Studies were excluded if other performance measures were used (e.g., return on investment, firm value). We focused on ROA, ROE, and Q, because they are relatively objective and the most commonly used indicators of organizational performance. Return on investments and firm value were not used, because there might be large differences in these measures due to economic differences between countries or due to strategic orientations. Unlike in Post and Byron [4], studies were excluded from our meta-analysis if female representation was measured as a dichotomous variable (presence vs. absence of females on board), or if they used measures that did not explicitly target the representation of females (e.g., the Blau Index or the Shannon Index), because they might bias the analysis. We also excluded studies that assessed female representation in an inappropriate body (such as the management or owners). This approach allowed us to achieve a reliable measure of female participation on corporate boards.

### Definition of variables

#### Firm performance measures

Firm performance was measured with three variables. First, ROA is computed by dividing the “earnings before extraordinary income and preferred dividend in financial year” by the “average of book values of total assets at the beginning and at the end of [a] financial year” [40]. The ROA measures the company’s production of “accounting based revenues in excess of actual expenses from a given portfolio of assets measured as amortized historical costs” [18]. Second, ROE is computed by dividing the company’s “earnings before extraordinary income and preferred dividend in [a] financial year” by the “average of book values of common equity at the beginning and at the end of a financial year” [40]. Thus, this measure assesses the returns to the company’s shareholders. ROE and ROA share a characteristic in that they are both “backward looking” accounting-based measures, meaning that they are based on the company’s self-reported financial performance in the recent past [40]. Third, Tobin’s Q is a market-based firm performance index calculated by dividing the firm’s “year-end market capitalization and average of book values of total debt at the beginning and at the end of [a] financial year” by the “average of book values of total assets at the beginning and at the end of [a] financial year” [40]. This measure is increasingly used in diversity research and scholars argue that it is more reliable than accounting-based measures [41], because it represents a “forward looking” measure, meaning that it reflects the future potential of a firm’s performance [40]. Beyond this, it is a standardized measure with intuitive interpretation criteria: If the ratio is greater than one, the firm has a higher ability to create value by effectively allocating its resources, indicating a high competitive advantage for that firm [42]. In contrast to ROE and ROA, this measure lacks an accounting convention bias, because it is considered objective by not relying on self-reported data [41].

#### Female Representation

Female representation was measured as the percentage of females on corporate boards.

#### Moderator variables

In addition to performance measures and female representation on boards, the following variables were included in the current analysis due to the relevant data in all, or most, of the studies:

1. *Country development and income*. Using the countries in which data were collected, we classified studies into dichotomous groups based on their economic development status (developed: *k* = 9; developing: *k* = 10) and their Gross National Income per capita (GNI; high income: *k* = 13; low income: *k* = 6; [43]).
2. *Mean board size*. This variable was a scale measure of the mean number of directors on boards.

### Data extraction

Data were extracted independently by three authors of this study from *k =* 20 studies (Post and Byron [4] included 13 of the 20 studies from our sample in their analysis), resulting in 34 coded effect sizes, and any inconsistencies were resolved during discussions. The authors of 16 studies were contacted via e-mail by the third author and seven provided additional data (either the sample size used for correlations or the correlation coefficients if they were not reported). Table 2 lists the study characteristics and effect size data.

**Table 2:** Study characteristics and effect size data in 20 studies included in the meta-analysis.

Study	Period	Data Source	Country	DEV/DC	GNI	Performance Measure	Mean (SD)	% Female	r	N	No. Firms	Board
[44]	2007–2011	Annual reports of all commercial banks	Kenya	DC	LI	ROA	—-	—-	.4760	45	9	—-
[44]	2007–2011	Annual reports of all commercial banks	Kenya	DC	LI	ROE	—-	—-	.0940	45	9	—-
[45]	2001–2009	Publicly limited firms on the OSE	Norway	DEV	HI	Q	1.55 (—-)	24.58	-.0665	1074	248	5.48
[46]	2000–2009	Publicly limited Norwegian firms	Norway	DEV	HI	ROA	—-	17.00	.0290	1560	~274	5.60
[47]	2003–2007	Publicly limited firms in Norway	Norway	DEV	HI	ROA	4.00 (17.00)	14.55	.0898	1279	128	7.56
[48]	2007	Public firms listed on the ISE	Indonesia	DC	LI	ROA	3.68 (7.12)	12.00	-.0700	169	169	8.44
[48]	2007	Public firms listed on the ISE	Indonesia	DC	LI	Q	2.08 (5.08)	12.00	-.1600	169	169	8.44
[49]	2005–2007	Australian Securities Exchange	Australia	DEV	HI	ROA	8.24 (6.29)	9.00	-.0600	151	151	7.58
[49]	2005–2007	Australian Securities Exchange	Australia	DEV	HI	ROE	15.27 (10.59)	9.00	.2200	151	151	7.58
[50]	2004–2009	Insurance firms listed on NSE	Nigeria	DC	LI	ROA	—-	9.51	.1389	72	12	9.06
[50]	2004–2009	Insurance firms listed on NSE	Nigeria	DC	LI	ROE	—-	9.51	.1635	72	12	9.06
[50]	2004–2009	Insurance firms listed on NSE	Nigeria	DC	LI	Q	—-	9.51	.0460	72	12	9.06
[40]	2001–2005	FTSE 100	UK	DEV	HI	ROA	—-	8.44	.0400	486	97	11.30
[40]	2001–2005	FTSE 100	UK	DEV	HI	ROE	—-	8.44	.0300	486	97	11.30
[40]	2001–2005	FTSE 100	UK	DEV	HI	Q	—-	8.44	-.1100	486	97	11.30
[51]	2008–2009	Firms listed on the Bursa Malaysia	Malaysia	DC	HI	ROA	3.41 (—-)	10.62	-.0150	280	280	7.63
[33]	1995–2004	MFI in Ghana	Ghana	DC	LI	ROA	39.16 (152.43)	37.38	.0111	520	52	6.23
[52]	2055–2007	Firms listed on the AEX	Netherlands	DEV	HI	ROE	14.91 (—-)	4.02	.3280	297	99	7.77
[9]	2007	Publicly listed firms in Mauritius	Mauritius	DC	HI	ROA	—-	3.10	.3370	42	42	9.60
[10]	1997–2011	Publicly traded Bank Holding Companies	USA	DEV	HI	ROA	4.65 (1.82)	7.94	-.1400	2640	212	12.68
[10]	1997–2011	Publicly traded Bank Holding Companies	USA	DEV	HI	ROE	9.92 (14.60)	7.94	-.1000	2640	212	12.68
[10]	1997–2011	Publicly traded Bank Holding Companies	USA	DEV	HI	Q	1.07 (0.07)	7.94	-.1500	2640	212	12.68
[53]	2004–2006	Spanish firms on the MSE	Spain	DEV	HI	ROA	—-	—-	-.0200	288	96	—-
[53]	2004–2006	Spanish firms on the MSE	Spain	DEV	HI	ROE	—-	—-	-.0250	288	96	—-
[53]	2004–2006	Spanish firms on the MSE	Spain	DEV	HI	Q	—-	—-	.0610	288	96	—-
[54]	2008–2012	Leading banks in Pakistan	Pakistan	DC	LI	ROA	3.10 (3.74)	33.30	-.0490	30	6	—-
[55]	2011	Firms listed on Bursa Malaysia	Malaysia	DC	LI	ROE	6.73 (11.27)	9.82	.0940	300	300	7.35
[11]	1998–2008	Agencies rating MFI	Worldwide	—-	—-	ROA	—-	—-	-.0039	497	329	—-
[11]	1998–2008	Agencies rating MFI	Worldwide	—-	—-	ROE	—-	—-	.0029	462	329	—-
[56]	2006–2007	Standard & Poor’s 500 Companies	USA	DEV	HI	ROA	0.00 (8.00)	14.00	.1000	185	185	10.48
[57]	2006–2010	Non-financial firms listed on the CSE	Sri Lanka	DC	LI	ROA	1.28 (0.83)	7.37	-.0112	440	88	7.33
[57]	2006–2010	Non-financial firms listed on the CSE	Sri Lanka	DC	LI	Q	—-	7.37	-.4300	440	88	7.33
[58]	2000–2009	Private listed firms on the CACM	China	DC	HI	ROA	3.81 (5.70)	12.33	-.0118	3197	~320	2.18
[58]	2000–2009	Privately listed firms on the CACM	China	DC	HI	Q	2.25 (1.47)	12.33	.0867	3197	~320	2.18

Abbreviations: AEX = Amsterdam Euronext Stock Exchange; ASX = Australian Securities Exchange; *Board* = mean size of the board; *CACM* = China’s A-Share Capital Market; *Country* = country of data collection; *CSE* = Colombo Stock Exchange; *Data* Source = Sampling Source of the studies*; DC* = developing country; *DEV* = developed country; *FTSE* = Financial Times Stock Exchange; *GNI* = Gross National Income Classification; *HI* = high-income; *IPO* = Initial Public Offering; *ISE* = Indonesian Stock Exchange; *LI* = low-income; *Mean (SD)* = mean (and standard deviation) of the performance measure; *MFI* = Microfinance Institutions; *MSE* = Madrid Stock Exchange; *N* = number of observations (number of firms × total length of data collection in years); *NSE* = Nigerian Stock Exchange; *No*. *Firms* = Number of firms in sample; *OSE* = Oslo Stock Exchange; *Period* = time frame in which data were collected; *% Female* = percentage of female board members.

### Data analysis (Meta-analysis)

The effect size used in the current meta-analysis was the Pearson product moment correlation coefficient, *r*. The interpretation criteria for the absolute magnitude of *r* are: .1 small, .3 medium, and .5 large [59]. The meta-analysis was computed using Comprehensive Meta-Analysis 2.0 (CMA; Biostat, USA) according to the random-effects model with inverse-variance weights [60]. The analysis was conducted in the following steps [60]:

1. The effect size data (*r* and *N*) were reported for each study. Since the variance of *r* is biased based on the magnitude of *r*, CMA converts *r* to Fisher’s *z*, computes all subsequent analyses on Fisher’s *z*, and converts the final results back to *r*.
2. Each effect size (Fisher’s *z*) was weighted according to the inverse-variance method (inverse of the sum of within- and between-study variance; [61]).
3. The overall mean weighted effect size of all studies was computed according to the random-effects model, where overall *r* is the sum of the product of all *r* (expressed as Fisher’s *z*) and weights divided by the sum of all weights. This model was used, because we assumed that the effect sizes would differ between studies in the analysis due to differences in study characteristics and because we only identified a random sample of all studies on this topic in our literature search.

The heterogeneity between study effect sizes was computed using a *Q* statistic and an *I* ^*2*^ index, where *I* ^*2*^ = 100%×(*Q*-*df*)/*Q* with *df* = *k*-1 and *k* = number of studies [60]. The *I* ^*2*^ index quantifies the variability in the effect sizes due to real (rather than chance) differences between studies. This variability can be interpreted as low (25%), moderate (50%), or high (75%) heterogeneity due to real differences between studies [62].

### Publication bias analysis

Studies with statistically significant and high effect sizes are more likely to be published in academic journals [60]. Such a publication bias could have inflated the result of our meta-analysis, which focused specifically on findings published in academic journals. Thus, we controlled for publication bias using methods available in CMA. Specifically, publication bias could be present if:

1. Rosenthal’s Fail Safe-*N* is low, meaning that it takes only a few theoretically missing studies with low effect sizes to nullify the result of a meta-analysis [63],
2. a funnel plot of standard error by Fisher’s *z* in each study is not symmetrical [64] and mathematically correcting for symmetry (using the trim-and-fill analysis) changes the interpretation of the overall analysis [65],
3. the study effect sizes and precision differ systematically according to the statistically significant Begg and Mazumdar correlation [66] and Egger’s regression [64].

### Sensitivity and moderator analyses

The stability of the overall mean weighted *r* over time was investigated by adding one study at a time to all previous studies (cumulative analysis). The influence of individual studies on the overall mean weighted *r* was investigated by removing one study at a time from the overall analysis (one-study removed analysis). The moderator analyses (subgroup analyses and univariate meta-regressions) were used to test the influence of systematic differences between studies on the overall mean weighted *r*.

## Results

### Study characteristics

A total of 20 studies with 34 effect sizes were included in the current meta-analysis. Firm performance was measured according to ROA in 85% of effect sizes (*k* = 17), ROE in 45% (*k* = 9), and Q in 40% (*k* = 8; Table 3). All 34 effect sizes were based on an average of 734 observations from 146 firms collected in slightly more than five years (Table 3). On average, the boards in those firms consisted of almost eight members with a low female representation (14%; Table 3). Half of all effect sizes were based on data from developing countries and data in 62% of the studies came from high income countries (Table 3).

**Table 3:** Descriptive statistics for *k* = 20 studies included in the current meta-analysis.

		*k* studies (% of 20)
WESP	# developing countries (% within outcome)	9 (45%)
	# developed countries (% within outcome)	10 (50%)
GNI	# high-income countries (% within outcome)	13 (62%)
	# low-income countries (% within outcome)	6 (32%)
Mean number of observations (*SD*)		734 (875)
Mean number of firms (*SD*)		146 (104)
Mean board size (*SD*)		7.89 (2.49)
Mean % female (*SD*)		13.82 (9.52)
Mean data collection period (*SD*)		5.32 (3.99)

*Note*. Abbreviations: GNI = Gross National Income per capita; Q = Tobin’s Q; ROA = Return on Assets; ROE = Return on Equity; *SD* = standard deviation; WESP = World Economic Situations and Prospects classification.

### Relationship between percentage of female representation and firm performance

**Fig 2:**
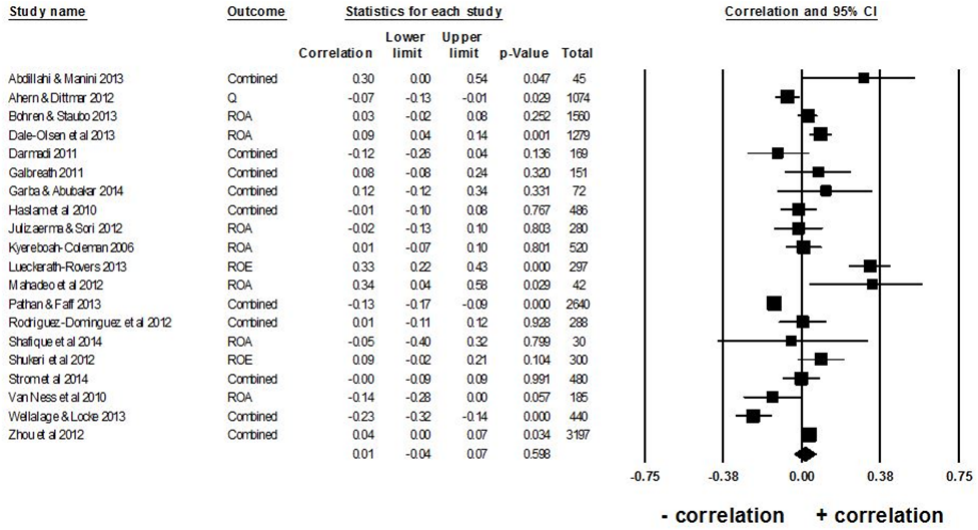
Forest plot of the association between percentage female representation on corporate boards and firm performance. ‘Correlation’ refers to the weighted Pearson product moment correlation coefficient, *r*. ‘Combined’ refers to the mean effect size in studies using multiple measures of firm performance. ‘Total’ refers to the total number of observations per study (number of firms × number of years). The diamond depicts the overall mean weighted effect size *r* of all *k* = 20 studies. There is a small positive, but not statistically significant, relationship between percentage female representation on corporate boards and firm performance (overall mean weighted *r* = .01, 95% confidence interval, *95%CI*:-.04, .07).

There was a small positive, but not statistically significant, relationship between the percentage female representation on corporate boards and the combined mean of the three firm performance measures; overall mean weighted *r* = .01, 95% confidence interval, *CI* [-.04, .07], *p* = .598, *k =* 20 (Fig 2). High variability in effect sizes among the 20 studies existed (*I* ^*2*^ = 87%, *Q* = 142.84, *df* = 19, *p* < .001).

### Publication bias analysis

**Fig 3:**
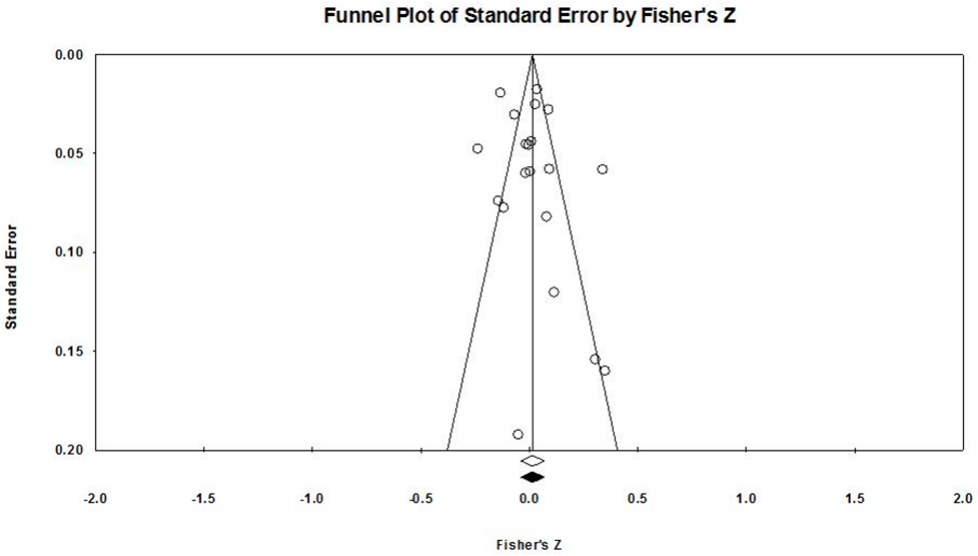
Funnel plot of the estimated variability (standard error of the mean, *SEM*) and effect size *r* (expressed as Fisher’s *z*) in each study. This plot shows that the effect sizes in the individual studies (circles) were symmetrically distributed around the overall mean weighted effect size shown on the vertical line.

Since the overall result of our analysis was not statistically significant, Rosenthal’s Fail-Safe *N* was not applicable. Nevertheless, there was little evidence of publication bias in the current analysis, because the funnel plot (Fig 3) was mathematically symmetrical around the central vertical line (corresponding to the overall mean weighted effect size) according to the trim-and-fill analysis. Thus, the overall mean weighted effect size in the analysis (unfilled diamond) and the overall effect corrected for potential missing studies (filled diamond) are aligned and no filled circles (theoretically missing studies) are shown on the plot (Fig 3). The symmetry is particularly evident in the area towards the top of Fig 3, showing the studies (unfilled circles) with the lowest estimated variability in effect sizes, and, thus, the highest precision. These studies contributed the most weights to the calculation of the overall mean weighted effect size in the current meta-analysis. Therefore, confirming the results on the forest plot (Fig 2), the studies with the most weights had either positive, negative, or close to null effect sizes, indicating that there was no preference for high positive, or high negative, effects in the current meta-analysis. Although the studies towards the bottom of Fig 3 appear less symmetrically distributed, these had high estimated variability of effect sizes, low weights, and, thus, little influence on the overall mean weighted effect size in the current analysis. Finally, study effect sizes and precision did not differ systematically according to the non-significant Begg and Mazumdar correlation coefficient (*p =* .770) and Egger’s regression intercept (*p* = .374).

### Sensitivity analysis

**Fig 4:**
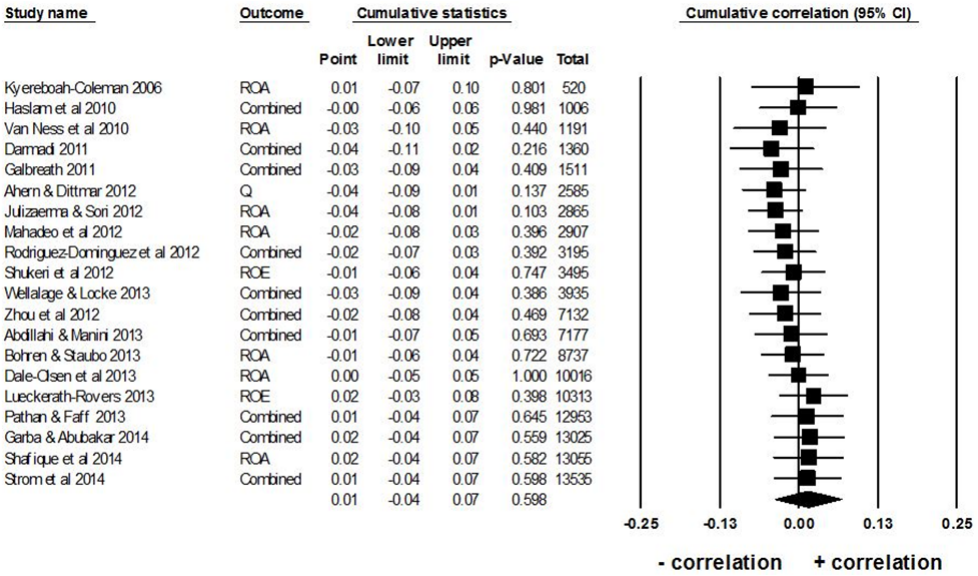
Forest plot of the cumulative analysis. ‘Combined’ refers to the mean effect size of studies that have used multiple measures of firm performance. ‘Total’ refers to the total number of observations (number of firms × number of years) as one study is added to all previous studies in each row. The plot shows how the overall mean weighted effect size *r* (referred to as ‘Point’) changes as each study is added over time to all previous studies. The diamond depicts the overall mean weighted effect size *r* of all *k* = 20 studies.

The cumulative analysis showed that the overall mean weighted *r* remained consistently small and non-significant as studies were added one at a time (based on publication date) from 2006 to 2014 (Fig 4).

**Fig 5:**
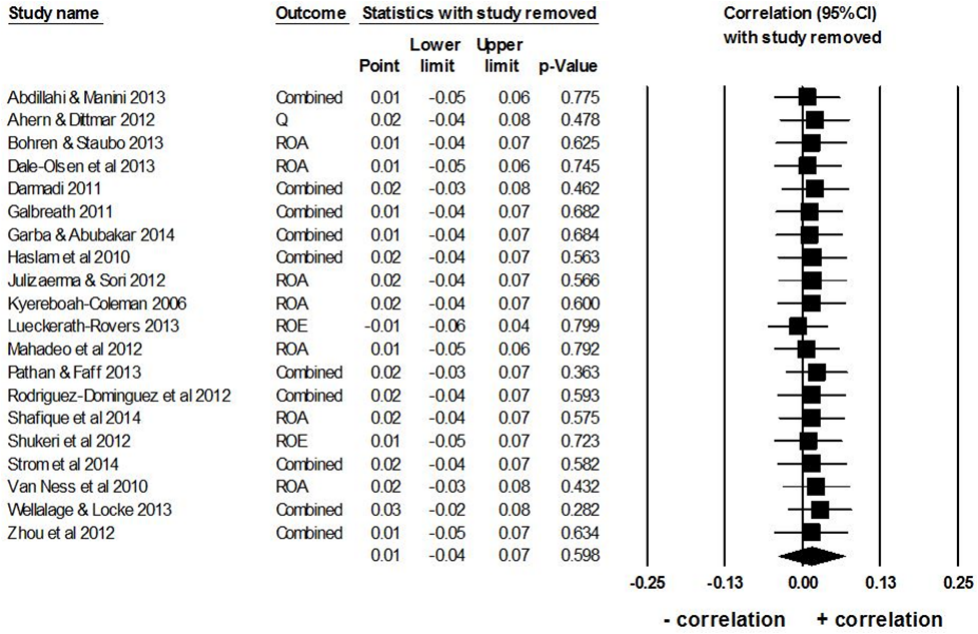
One-study removed analysis. Combined’ refers to the mean effect size from studies that have used multiple measures of firm performance. The plot shows the overall mean weighted effect size *r* (referred to as ‘Point’) for all studies, except the study in each row. The diamond depicts the overall mean weighted effect size *r* of all *k* = 20 studies.

Similarly, the overall mean weighted *r* was not influenced by any one study, because it remained small and non-significant as one study at a time was removed from the overall analysis (Fig 5).

### Subgroup analysis and meta-regression

According to a subgroup analysis, the overall outcome of the current meta-analysis was not dependent on the performance measure. Specifically, the overall mean weighted *r* remained consistently small and non-significant when studies were grouped according to individual performance indicators (Table 4).

**Table 4:** Results of the moderator analysis and the meta-regressions.

	Subgroups	*k* studies (% of 20)	*Mean weighted r (95%CI)*	*p*_*two-tailed*_
**Performance Measure**	ROA	17 (85%)	.00 (-.05, .05)	.861
	ROE	9 (45%)	.08 (-.02, .19)	.125
	Q	8 (40%)	-.10 (-.21, .02)	.107
**WESP Development**	Developed countries	9 (47%)	-.02 (-.06, .10)	.661
	Developing countries	10 (53%)	.01 (-.07, .10)	.753
**GNI**	Lower income countries	6 (32%)	-.02 (-.17, .12)	.750
	Higher income countries	13 (68%)	.03 (-.03, .10)	.310
	**Meta-regression predictor**	***k studies***	***Slope***	***Slope p***_***two-tailed***_
	Mean Board Size	16	-.01	.400

Similarly, the overall mean weighted *r* remained consistently small and non-significant when studies were grouped according to either country development (developed vs. developing) or country income (lower vs. higher income); Table 4.

Finally, as shown in Table 4, the univariate meta-regression showed that the weighted effect sizes in individual studies (outcome) were not dependent on the mean board size (predictor).

## Discussion

The main finding of the current study, based on data from 20 studies (34 effect sizes) published only in peer-reviewed academic journals, is that the relationship between the percentage of female directors on corporate boards and firm financial performance is consistently small and non-significant. The general magnitude of this result is in line with findings of Post and Byron [4], who, based on data from 140 published and unpublished studies, also found a small correlation between gender diversity on corporate boards and firm financial performance. This is especially noteworthy, because both meta-analyses are based on different study samples and different operationalizations of the main measures (female representation and firm performance), providing further evidence to conclude that female representation and firm performance are not strongly associated. Interestingly, the two meta-analyses differ in that Post and Byron [4] find a *statistically significant* correlation between increased gender diversity on corporate boards and higher accounting returns. But concluding that a higher female representation on corporate boards has practical implications for the generation of profits from assets and investments seems debatable due to the overall small effect size. By testing the relationship of interest with our more rigorous and controlled methodological approach (for example, by including only peer-reviewed and published studies), we provide further evidence that female representation on corporate boards is not associated, positively or negatively, with firm performance. Although both meta-analyses indicate only a very small correlation, primary research should further investigate the relationship between boards’ gender composition and firm performance. This is because, as argued below, the relationship might be too complex to be investigated on a univariate level.

In our international sample, female representation on corporate boards was not significantly related to firm financial performance, as measured by the “backward-looking” measures ROA and ROE and the forward-looking measure Tobin’s Q, if we did not control for any other factors. This result is in line with other primary studies and meta-analyses [3]. [57], [67], indicating only a small association between (gender) diversity and firm financial performance, while contrasting individual studies that find either a positive [9] or negative relationship [10] between gender diversity and firm performance.

Results of both meta-analyses provide little evidence to support the business case for gender diversity. However, more importantly, a higher representation of females on corporate boards is also not associated with a detrimental effect on firm financial performance, which supports the ethical case for diversity. If increased female representation on corporate boards is not positively or negatively associated with firm performance, it seems reasonable to promote gender equality in board representation. Given the current underrepresentation of females on corporate boards in all studies included in our sample, and possibly in all countries worldwide (the largest average percentage of female directors included in our sample was 37% [33] and the overall average was only 14%), women should be prioritized for promotions if they are equally qualified. By bringing the performance-based and the ethical view together, fostering gender diversity in boardrooms seems justified and desirable.

We do, however, acknowledge that our univariate approach to this intricate research question is rather simplistic and might not do justice to the vast econometric complexity present when studying the relationship between gender diversity and firm performance. Numerous other variables not investigated in this study might influence the relationship between female representation on corporate boards and firm financial performance. Future meta-analyses should investigate the relationship between various other diversity variables, such as age, tenure or education, on corporate boards and firm financial performance and subsume them in one analysis. Such an inclusive approach would yield benefits for practitioners and scientists.

The board’s limited influence on the firm financial performance might be a reason for the small overall effect size in the current meta-analysis. According to Bertrand and Schoar [68], the CEO and CFO only explain about 5 to 6% of variance in corporate performance measures. Hence, the boards’ effect on actual firm performance may be limited in general. Various other factors which the board cannot alter or influence, such as the current economic and political situation, influence companies’ performance. Although such factors might be more important for firm performance than gender alone, they are difficult to quantify numerically for meta-analytic purposes.

### When the business case might still matter

The business case for diversity should not be abolished altogether. Whether an increased representation of females and the concomitant increase in gender diversity on corporate boards lead to performance benefits for the firm might depend on contextual factors, or on how diversity is managed. In our analysis, we aimed to find moderators of this relationship based on systematic differences between studies. Not surprisingly, the process of finding specific moderators was difficult, because there was high heterogeneity among studies in terms of reported study characteristics which could be used as potential moderators.

The relationship between female representation and firm performance remained independent of how firm financial performance was measured. This supports our initial assumption that these outcome measures are relatively objective and measure firm financial performance similarly. In addition, this finding increases the certainty with which our results can be interpreted, because the non-significant relationship seems to be independent of the outcome measure. On a descriptive level, the results of this subgroup analysis are also in line with Post and Byron [4], who find that accounting returns, such as ROA and ROE, increase and market performance (Tobin’s Q) remains unrelated to whether there are more female directors on corporate boards. While the significant positive relationship between accounting returns and female representation on corporate boards, which Post and Byron [4] find, deviates from our nonsignificant finding regarding these performance measures, their large sample size, which increased the power of their analysis and the likelihood of finding a significant result, is likely the reason for this deviation. Regardless of statistical significance, the overall magnitude of their effect size for ROA and ROE was similar to our effect sizes, and deducing practical implications from such small effect sizes might be debatable and misleading.

The characteristics of the country in which data were collected in the individual studies had little influence on the effect sizes in the current analysis. Neither a country’s development status, nor its GNI per capita influenced the relationship between female representation and firm financial performance. Thus, whether a country is considered developed/developing or “poor”/“rich” does not influence the effect that female board members have on firm performance. While previous studies have shown that a country’s characteristics, such as a long history of female participation in politics [69], influence the presence of women on corporate boards, our results suggest that a country’s characteristics related to economic performance do not influence the relationship between female representation and firm financial performance. This indicates that aiming for equality should guide decisions regarding future promotion to corporate boards, irrespective of a country’s economic status.

In addition to our more rigorous meta-analytical approach to the research question, we also tested what Post and Byron [4] called for: the moderating effect of board size on the relationship between female representation and firm performance. Larger boards might make it more difficult for directors to influence decisions and might limit the influence of directors on important decisions overall [70], [71], and the percentage of female directors on larger boards would therefore also matter less. However, the number of directors on corporate boards did not significantly influence the effect size distribution in our analysis, suggesting that the non-significant relationship between female representation and firm performance remains similar, regardless of how many directors are on corporate boards, at least on the meta-analysis level. This is somewhat surprising, because larger boards usually experience a higher complexity in all decision-making processes [72] and have been shown to be associated with decreased financial performance (ROE; [73]). The increased complexity on larger boards might make it even more difficult for females to have an impact, given their apparently wide underrepresentation. In conclusion, although a higher representation of females on boards does not appear to be directly associated with financial performance, more females on corporate boards might indirectly influence firms’ financial performance. For example, females might provide a protective effect against larger boards’ apparently increased interpersonal conflicts. However, this requires further research.

### Limitations and Future Research

There were a number of limitations in the current study. First, the main limitation is the small sample size of only 20 studies in our analysis, making it difficult to accept the null hypothesis due to low statistical power. However, overall, Post and Byron [4] also report a similar result based on the higher number of studies in their analysis. Thus, it is unlikely that the apparent lack of a meaningful relationship between female representation on corporate boards and firm performance just resulted from low statistical power in both meta-analyses. Interestingly, regardless of the studies’ high heterogeneity, the variability of the overall mean weighted effect sizes is reasonably consistent in our analysis. Specifically, as shown on Fig 2, the effect sizes in all 20 studies can be classified as either positively or negatively small (most weighted correlation coefficients between ± .3). Thus, although it cannot be ruled out, it is unlikely that statistical power alone, or one or more hidden factors, consistently suppressed a relationship between female representation on boards and firm financial performance based on data from different countries and various industries. Future primary studies should attempt to focus on other diversity factors, such as educational level or the seniority of female board members of successful companies to determine how these characteristics affect firm performance.

Second, the quality of the included studies was not assessed by means of standardized scales. Instead, we only selected studies published in academic journals, assuming that such studies are of a higher quality than unpublished sources, because experts had reviewed them. Furthermore, we also assumed that academic research on the topic might be more value-free than those in unpublished sources. Our assumption might not have been entirely correct, because the quality of the reported statistical data was generally poor in many of the examined studies. For example, although most studies conducted complex multivariate statistical analyses, it was often unclear if the reported regression coefficients were unstandardized or standardized (meaning that high-quality multivariate data corrected for various factors could not be used in our analysis). Therefore, the authors of future primary studies should use standardized guidelines to report quantitative results, which can be subsequently utilized in meta-analyses.

Third, including only published sources could potentially lead to a publication bias (inflation of effect sizes) in a meta-analysis. However, based on the outcome of the various tests we conducted, there was little evidence of publication bias in our analysis. Interestingly, in the current analysis, the strongest evidence against publication bias is a simple visual inspection of the data in Fig 2. All the effect sizes in this figure are small, suggesting that the opposite of publication bias might have occurred in this analysis. That is, we might have failed to find studies with *high* magnitude effect sizes in the positive or negative direction.

Fourth, the current study relies on a linear model to determine the relationship between female representation and firm financial performance. It is possible that such a relationship depends on multiple factors in a non-linear fashion and that this changes over time. Thus, the linear assessment of data collected over a number of years might have contributed to the low effect sizes in the current meta-analysis. In general, it is difficult to determine the correct analytical approach to such a complex topic. Specifically, the relationship between female representation and firm financial performance was low, not only on a univariate study level, but also after the application of multivariate linear and non-linear approaches in the primary studies included in our analysis. Thus, the relationship between gender and financial performance might be truly negligible compared to other factors that might affect financial performance. Thus, future studies should focus on devising novel analytical approaches to study this topic.

Fifth, the data in our analysis come from countries with differing legal and board systems. Most pronounced is the difference between the one-tier and the two-tier board system, with various smaller differences between countries. In a one-tier board system, which is prevalent in the United States, the board is solely responsible for all corporate decisions. Inside directors, who are directly employed in the company, represent the interests of the company’s stakeholders, while outside directors, who are usually employed in other companies, bring a different perspective and objectivity to the boardroom. A two-tier board system, which, for example, is common in Germany, consists of an executive board and a supervisory board. The executive board manages the day-to-day business and the supervisory board supervises the executive board’s decisions. We did not investigate the influence of these factors, because there are too many minor differences between countries and they are too widespread to classify. While we do not expect these country differences to have a large influence on the current results, they might have contributed to the high heterogeneity in the studies’ effect sizes. Meta-analytically investigating the differing role that the diversity of internal and external directors might play in the boardroom with regards to firm performance could provide a future contribution to the scientific literature.

Sixth, two studies included in our sample were classified as statistical outliers due to their extreme effect sizes in opposite directions [44], [57]. The removal of these studies did not change the outcome of our analysis. These studies possibly were outliers, because both included a relatively small number of firms, nine and 88, respectively (compared to the mean number of firms of 146 in all the studies; see Table 3). This might have increased the influence of individual firms and thereby skewed those studies’ results.

Seventh, it is possible that not all 20 studies were based on independent data, meaning that some firms might have contributed more data to the overall effect size than others. There was a possible overlap in the included firms from the same countries, because the primary studies did not rely on single firm data, but rather on data from business databases from the same country. For example, data from Norway were included in three studies in the current analysis [45]-[47]. To reduce this potential overlap in the data, we only once included data from the same time period, utilizing the same outcome measure in the same country.

Eighth, an overall higher representation of females might be needed in order to identify a relationship between diversity and performance. A shortcoming of the included data is that they are restricted in range, because the largest percentage of female directors included in our sample was 37% and the average was about 14%. This limits the meaningfulness of our findings, because few female directors are present in all the studies in general. This result supports the notion that more females should be promoted to director positions to meaningfully investigate the effects of gender diversity on performance. In accordance with this proposition, Joecks et al. [74] suggest that a relatively low representation of females on boards first has a negative effect on firm performance (contradicting our findings), which only becomes positive after a critical mass of 30% female directors is reached. Thus, the current representation of 14% females on corporate boards in our sample might not be sufficient to show either the positive or negative effect that increased gender diversity might have on firm performance.

Lastly, women generally experience higher levels of chronic stress than men [75], especially when working in male-dominated industries, where they suffer from increased stress and worse mental health than men [76] and might be perceived as tokens [36]. These differences in experienced stress might influence the relationship between gender diversity and firm performance, depending on the industry in which it is measured. For example, it would be interesting to examine if women’s influence is more pronounced in traditionally female-dominated industries as opposed to male-dominated industries. Similarly, Post & Byron [4] also suggest that female directors might influence firm performance stronger in customer-proximal industries.

## Conclusion

In recent years, interest in gender diversity and the representation of females in leadership positions has evidenced a steep increase. Many scientific studies have investigated the relationship between female representation on corporate boards and firm financial performance, but, so far, the results are contradictory. The results of the current meta-analysis show that a higher representation of females on corporate boards is neither related to a decrease, nor to an increase in firm financial performance, confirming findings from a similar meta-analysis on this topic [4]. These results do not support the business case for diversity, which suggests that diversity is associated with an increase in performance. However, they allow the conclusion that gender diversity should be promoted for ethical reasons to promote fairness. If a larger representation of female directors does not matter with regard to firm performance, females, if equally qualified, should be given priority when promotion decisions are made.

## Supporting Information

S1 PRISMA Checklist (DOC)
